# Corrosion of Bronzes by Extended Wetting with Single *versus* Mixed Acidic Pollutants

**DOI:** 10.3390/ma7053353

**Published:** 2014-04-28

**Authors:** Liliana Gianni, Giovanni E. Gigante, Mauro Cavallini, Annemie Adriaens

**Affiliations:** 1 Department of Analytical Chemistry, Ghent University, Krijgslaan 281-S12, 9000 Ghent, Belgium; 2 Dipartimento di Scienze di Base e Applicate all’Ingegneria, Università di Roma La Sapienza, Via A. Scarpa 14/16, 00161 Rome, Italy; E-Mails: liliana.gianni@uniroma1.it (L.G.); giovanni.gigante@uniroma1.it (G.E.G.); 3 Dipartimento di Ingegneria Chimica Materiali Ambiente (DICMA), Università di Roma La Sapienza, Via Eudossiana 18, 00184 Rome, Italy; E-Mail: mauro.cavallini@uniroma1.it

**Keywords:** bronze corrosion, tin influence, acid rain, electrochemical impedance spectroscopy, spectrocolorimetry

## Abstract

The corrosion of bronzes was examined in the context of single-acid *versus* mixed-acid (as in urban acid rain) solutions. Two bi-component bronzes (copper with either 3% Sn or 7% Sn) that closely represent those of historic artifacts were immersed for five weeks in conditions designed to replicate those experienced by statues and ornaments in cities where rainfall and humidity constantly produce an electrolyte layer on the surfaces of bronzes. Ions, acids, and particles of pollutants can dissolve in this layer, resulting in a variety of harsh corrosion processes. The kinetics of corrosion and the properties of the resulting patinas were monitored weekly by electrochemical impedance spectroscopy and open-circuit potential measurements. The sizes and appearances of the corrosion products were monitored and used to estimate the progress of the corrosion, whose crystalline structures were visualized using scanning electron microscopy with energy dispersive spectroscopy, identified by X-ray diffraction, and characterized by spectrocolorimetry. The electrochemical measurements demonstrated that greater damage (in terms of color change and corrosion product formation) did not correspond to deficiencies in protection. The mixed-acid solution did not corrode the bronzes, as would be expected from the additive effects of the single acids. The postulated mechanisms of metal dissolution appear to be specific to a particular bronze alloy, with the tin component playing an important role.

## 1. Introduction

Corrosion is a complex process involving many factors. Composition, morphology, and surface inclination are the main factors affecting the corrosion of an object [1–9]. Environmental parameters, such as humidity, temperature, rain, and air quality, also play a role [10–16]. Once formed, the corrosion products themselves also directly influence the process. The complexity and specificity of corrosion mechanisms arise, therefore, on account of combinations of multiple factors [17–20].

A comprehensive understanding of the various different corrosion mechanisms is important to the protection of cultural artifacts. Investigations have been conducted during the analysis of degraded monuments undergoing restoration [21–27], and various relationships have been identified between the compositions of artifacts, their level of corrosion, and the monument environment, e.g., indoor, outdoor, urban, marine, or rural [28–33].

Given that a great many monuments are exposed to aggressive urban pollution, studies of corrosion with respect to conservation are receiving increased research attention [11,13,15–18,34,35]. Although air pollution levels can be mitigated by legislative regulation, the problem of corrosion remains large.

In this study, we examined the corrosion of bronzes with the aim of understanding reaction mechanisms. The following factors were considered: alloy composition (two bi-component alloys were tested), composition of the aqueous layer adsorbed on the bronzes, effects of acid pollutants (*i.e*., applied singly and in combination), and the duration of wetting. The two alloys were chosen to be representative of bronze artifacts exposed across Europe. Ornamental bronzes generally contain less than 10% Sn. Acid rain was simulated using solutions of its main acidic components (sulphuric and nitric acids).

## 2. Results and Discussion

### 2.1. OCP Monitoring

**Figure 1.**
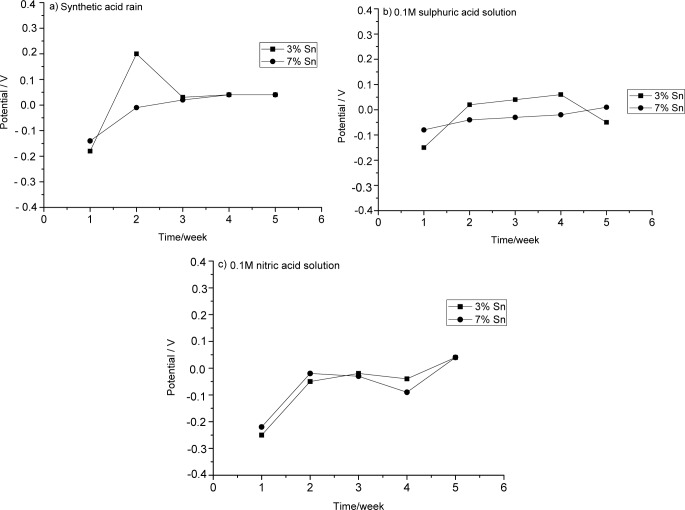
OCP measurements during five weeks of immersion of 3% Sn and 7% Sn bronzes in (**a**) synthetic acid rain solution; (**b**) 0.1 M sulphuric acid; and (**c**) 0.1 M nitric acid.

The evolution of OCP during five weeks of immersion in the three aggressive solutions is shown in Figure 1.

#### 2.1.1. OCP Measurements of Bronzes Immersed in Artificial Acid Rain Solution

The bronze with 3% Sn was initially more greatly corroded than the other alloy. The decrease of OCP suggests that its patina was less protective. (Cicileo *et al.* noted that more stable and protective patinas show high and constant potential values [19].)

The bronze with 7% Sn showed a slow but continuous patina formation, as demonstrated by the slight increase of OCP toward anodic values. The potential appeared to be almost stable from the third week to the end of the five-week observation, suggesting the emergence of a stable, protective layer.

#### 2.1.2. OCP Measurements of Bronzes Immersed in Sulphuric Acid Solution

During its first week in sulphuric acid, the 3% Sn bronze showed a great increase of potential owing to patina formation. This patina remained almost stable, and thus protective, until the fourth week, when it partially dissolved as the aggressive solution penetrated through cracks in it.

The 7% Sn sample formed a more stable patina.

#### 2.1.3. OCP Measurements of Bronzes Immersed in Nitric Acid Solution

The 3% Sn bronze formed a patina in two anodic steps during the first and the fourth weeks. Between these two steps, the patina, and, thus, the potential, remained stable. The 7% Sn bronze showed an anodic reaction during the first week, a slight decrease of its potential during the second week, and an even stronger decrease in the third week. After this, a stable patina re-formed. The trends of the OCP curves of the two samples are comparable, and their potential values also appear to be similar to each other.

### 2.2. Electrochemical Impedance Spectroscopy

#### 2.2.1. EIS of Bronzes Immersed in Artificial Acid Rain Solution

The equivalent circuits of the 3% Sn bronze reflect the formation and dissolution of its patina. During the first week, the equivalent circuit evolved from *R*_e+ct_*Q* (which considers the sum of the electrolyte resistance and the charge transfer resistance) to *R*_e+ct_*QW* (Table 1); it then changed to *R*_e_(*R*_f+ct_*Q*)*W*, which indicated a higher resistance of charge transfer (with a partial contribution of the film resistance) due to the growth of a monolayer patina. The values of n and W indicate that the layer was rough and porous and allowed diffusion phenomena (Warburg, *W*).

The re-forming of the patina after its partial dissolution can be implied from the calculated circuit elements: *R*(*R*_f_*C*_f_)(*R*_ct_*Q*)*W* (*R*_f_ and *C*_f_ are related to the corrosion layer properties, while *R*_ct_ is the charge transfer resistance). The charge transfer capacity (*Q*) and the Warburg (*W*) underline that charge transfer and diffusion processes respectively dominated the reaction. The *Q* index (*n* = 0.38) also indicates the roughness of the layer that allowed diffusion inside the layer, as also reported in other studies [29–36].

The mechanism of the charge transfer continued until the end of the fourth week. The resistance increased from 9.94 Ω in the third week to 1.17 × 10^3^ Ω in the fourth week, and the capacitance decreased from 4.91 × 10^−4^ F in the third week to 6.9 × 10^−6^ F in the fourth. These changes can be interpreted as an improvement of the patina’s passivity. During the last week, the resistance reached 4 × 10^7^ Ω.

The 7% Sn bronze developed different equivalent circuits. During the first week, diffusion, represented by Warburg (*W*), and charge transfer (*R*_f+ct_*Q*) reactions were active on the surface (*R*_e_(*R*_f+ct_*Q*)*W*). In the second week, the surface was identified by a two-circuit mesh: *R*_e_(*R*_f_*Q*)(*R*_f+ct_*Q*)*W*, which accounted for the protective properties despite the porosity (*W* and *n* are both present in the expression representing the roughness of the surface and the diffusion). During the third week, the equivalent circuit was composed only of *R*_e_(*R*_f+ct_*Q*), indicating that the dissolution of the outer layer (the second mesh of the equivalent circuit of the second week) occurred due to its porosity—the *n* index of the outer layer was 0.54, and that of the inner layer was 0.76. The less rough and more homogeneous inner layer (*n* = 0.76) was more stable and passive than the outer one (*n* = 0.54); it underwent aggressive kinetic processes attributable both to charge transfer and diffusion (*W*). However, the mechanism of charge transfer inside the layer caused the patina to grow, which reduced the quantity or dimensions of the pores. In this way the film showed a higher resistance (*R*), implying better protective properties.

The patina growth then slowed the corrosion progress, and the circuit went from (*R*_e+f_*Q*)(*R*_ct_*C*_ct_) during the fourth week, when the process was determined by the patina formation, to *R*_e_(*R*_f+ct_*Q*)*W* during the fifth week.

The relative thickness of the 3% Sn bronze was calculated using the equivalent circuit of the second week (the equivalent circuit in which the film resistance is distinguishable from the other resistances). Its high value of 8.47 × 10^5^ F^−1^ can be attributed to the roughness and porosity of the layer. The *n* index was 0.38, and Warburg diffusion was also present. This interpretation was confirmed by the subsequent partial dissolution of the patina in accordance with the progression of the OCP and the relative equivalent circuit.

During the fourth week, the relative thickness of the patina on the 7% Sn bronze reached 7.29 × 10^3^ F^−1^. The patina was thinner but more stable than that grown on the 3% Sn sample, as also demonstrated by the OCP and the EIS results.

Overall, the 7% Sn bronze produced a double layer of corrosion products, wherein the dissolution of the outer film improved the barrier properties of the inner one. The 3% Sn sample appeared to be the more damaged, with a thick but unstable patina.

#### 2.2.2. EIS of Bronzes Immersed in Sulphuric Acid Solution

Table 2 lists the equivalent circuits and the values of each element for the two alloys immersed in sulphuric acid. The 3% Sn bronze changed from an *R*_e_*Q* equivalent circuit on the first day of immersion to an *R*_e_(*R*_f_*C*_f_)(*R*_ct_*C*_ct_)W equivalent circuit after one week. This underlines the rate of the initial corrosion process. However, the patina allowed diffusion (*W*). During the second week, the patina continued to form, but more slowly than in the first week, as shown by the sum of the film and charge transfer resistances (*R*(*R*_f_*C*_f_)(*R*_ct_*W*)). The resistance increased until the fourth week. The subsequent partial dissolution again exposed the surface to the aggressive solution.

Patina formation was more progressive on the 7% Sn sample. However, the equivalent circuits outline the different phenomena acting during the test. The fast corrosion of the first week, represented by the increase of *Q* values, indicates a preference for the charge transfer process. The *n* index (that is present only with the *Q* element) of 0.42 indicates the roughness of the film. Charge transfer in the film distinguishes the equivalent circuit of the second week. Diffusion, as Warburg (*W*), also occurred in the following equivalent circuit: *R*_e_(*R*_f_*C*_f_)(*R*_ct_*Q*)*W*. The patina feformation from the third to the fifth week can be expressed by a *R*_e+f_(*R*_ct_*Q*) circuit. A diffusion mechanism occurred only during the fourth week; the values indicate a decrease of the resistance and an increase of the capacitance. This demonstrates that corrosion increased owing to the porosity of the patina and its partial dissolution. The relative thickness of the patina of the 3% tin sample was calculated from the circuits of the first, second, and fourth weeks. It was 2 × 10^3^ F^−1^ in the first week, increasing to 3.4 × 10^5^ F^−1^ in the second week and 4.76 × 10^5^ F^−1^ in the fourth week. The results are compatible with the interpretation of the OCP and EIS results. The relative thickness of the patina of the 7% tin sample in the second week was higher: 8.3 × 10^5^ F^−1^. Overall, these results also demonstrate the good corrosion behavior of the 7% Sn sample, which showed progressively decreasing corrosion due to the formation of a stable patina.

#### 2.2.3. EIS of Bronzes Immersed in Nitric Acid Solution

The 3% Sn bronze corroded quickly until the second week; its equivalent circuits for the first two week can be represented as *R*_e_(*R*_f+ct_*Q*) (Table 3). The resistance increased and the capacity decreased, indicating that patina formation improved its passivity, despite the porosity of the layer. During the third week, this trend reversed, and a W term appeared (*R*_e_(*R*_f_*Q*)(*R*_ct_*Q*)*W*): charge transfer and diffusion both occurred on the surface. These reactions involved a more stable and less porous patina than was present the previous week; it reached its highest resistance values in the last week. This analysis is comparable with the OCP results.

The relative thickness of the patina during the fifth week for the 3% Sn sample was 1.16 × 10^6^ F^−1^; that of the 7% Sn sample was 1 × 10^4^ F^−1^. The film growth could not be calculated for the following week because the circuit elements were formed by the sum of the resistances (*R*_Tot_ = *R*_e_ + *R*_f_ + *R*_ct_).

A more stable and protective patina appeared to form on the 3% Sn sample in this test; it was comparable to the less passive layer of the 7% Sn sample.

### 2.3. Spectrocolorimetry

**Figure 2.**
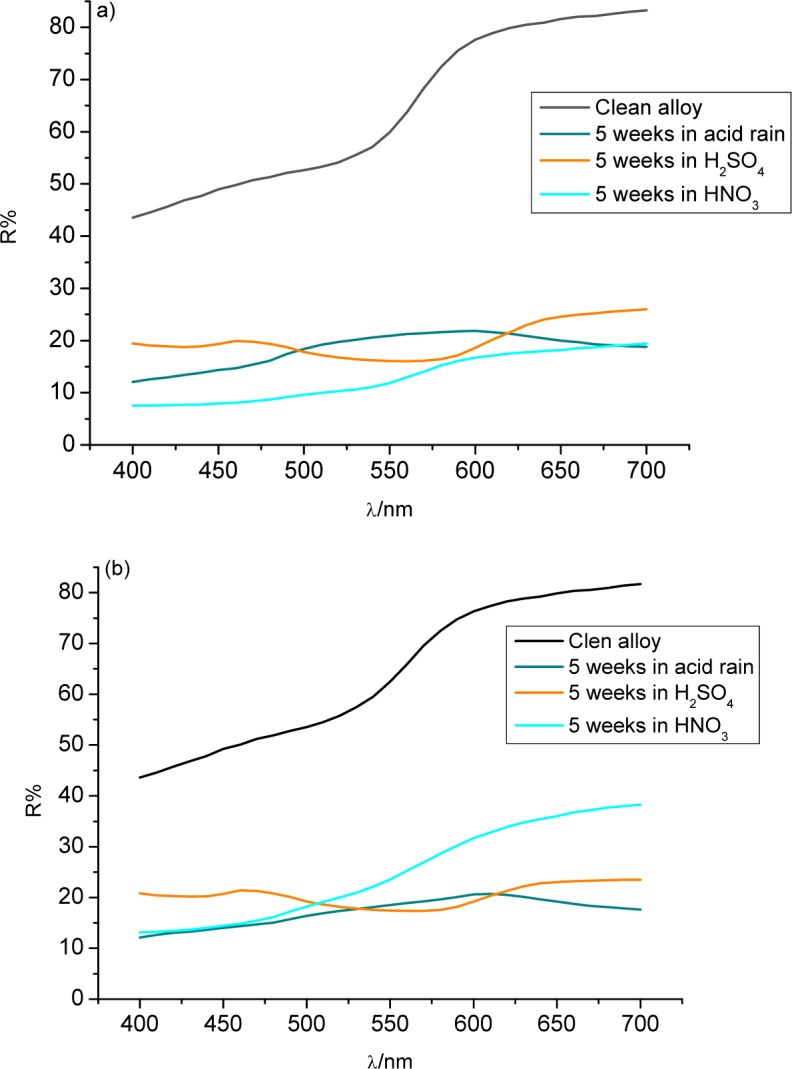
Reflectance curves of (**a**) 3% Sn bronze and (**b**) 7% Sn bronze after five weeks of immersion in artificial acid rain, 0.1 M sulphuric, and 0.1 M nitric acid.

Figure 2 shows the reflectance curves for each alloy after five weeks of immersion. Both alloys emerged from the acid rain solution looking green, hence the peaks in the 500–600 nm region. Immersion in sulphuric acid created a purple patina on each alloy, which each showed a peak on its reflectance curve at around 450 nm.

Nitric acid caused both alloys to show greatly reduced reflectance, particularly the 3% Sn sample. The 3% Sn sample showed its greatest loss of reflectance in nitric acid, while acid rain caused the greatest loss for the 7% Sn alloy. This demonstrates the different behaviors of the alloys in different environments.

**Figure 3.**
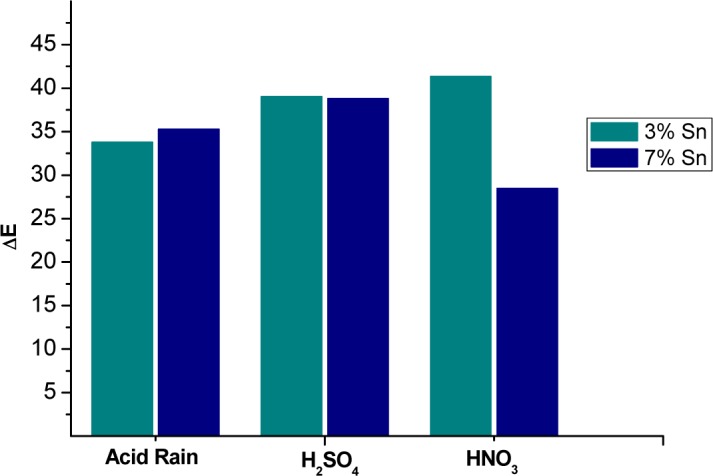
Color difference (Δ*E*) between the clean surfaces of 3% Sn bronze and 7% Sn bronze and after their immersion for five weeks test in synthetic acid rain, sulphuric acid, and nitric acid.

The Δ*E* values (Figure 3a, and Table 4) show in detail the changes: the greatest color difference for the 3% Sn alloy (mainly a decrease of lightness L*) was due to nitric acid. A high and comparable Δ*E* for the 3% Sn bronze was obtained in sulphuric acid for the relevant changes of all the color parameters (L*, a* and b*) (Table 4). Important parameter variations were also detected in the acid rain test, but the color difference (Δ*E*) was smaller.

The 7% Sn bronze showed a large color difference upon its immersion in sulphuric acid (its yellow–blue component b* index varied greatly), while the acid rain solution greatly affected the a* index (red–green component) (Table 4).

The Δ*E* values show that the 3% Sn sample underwent greater color changes than did the 7% Sn bronze in each acidic environment, except for the acid rain solution, in which the 7% Sn sample was more damaged.

### 2.4. SEM–EDS and X-ray Diffraction Spectroscopy

**Figure 4.**
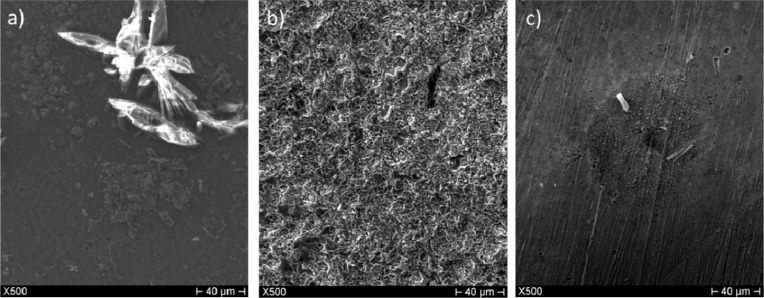
SEM secondary electron images at ×500 for the 3% Sn sample immersed for five weeks in (**a**) synthetic acid rain solution; (**b**) 0.1 M sulphuric acid; and (**c**) 0.1 M nitric acid.

Figure 4A shows secondary electron images of the 3% Sn bronze immersed in acid rain. The white crystals are sulphur compounds grown on a layer of oxides products. The smaller dark crystals are chloride compounds. The 7% Sn showed a similar patina.

The patina formed in sulphuric acid solution appeared to be very different. Large crystals grew homogeneously on the surface of the alloys (Figure 4B). Elemental composition assessment revealed the presence of copper and oxygen, which were likely present as cuprite (Cu_2_O) and/or tenorite (CuO). The patina formed in nitric acid was less developed; it appeared inhomogeneous with isolated nuclei of corrosion products (Figure 4C).

**Figure 5.**
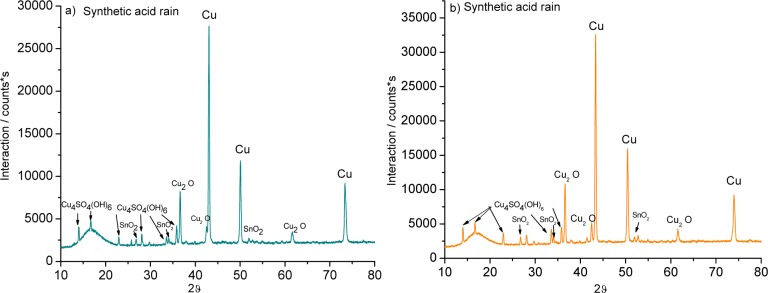

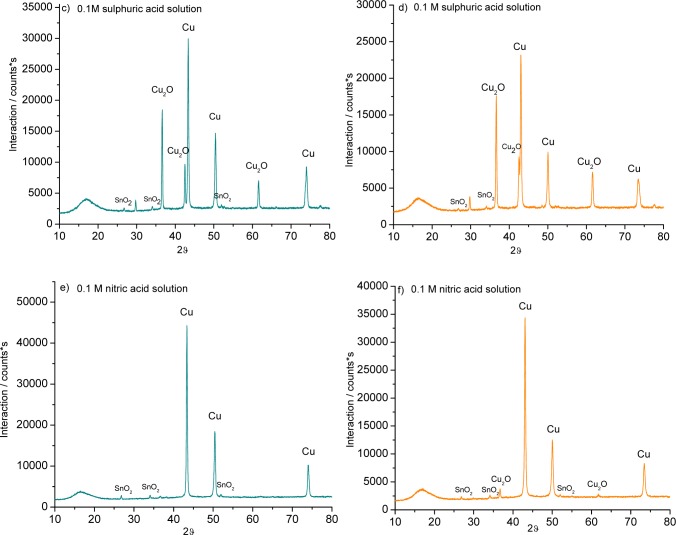
XRD patterns of (**a**,**c**,**e**) 3% Sn bronze and (**b**,**d**,**f**) 7% Sn bronze in (**a**,**b**) synthetic acid rain; (**c**,**d**) 0.1 M sulphuric acid; and (**e**,**f**) 0.1 M nitric acid.

The XRD, performed on the bronze pieces, support the chemical analysis and the interpretation of the EDS results (Figure 5). The patina was formed of oxides such as cuprite (Cu_2_O) and cassiterite (SnO_2_) and from a sulphur corrosion product known as brochantite (Cu_4_SO_4_(OH)_6_).

The patina formed on both alloys in sulphuric acid was simpler than the layer formed in the mixed-acid solution: cuprite (Cu_2_O) and cassiterite (SnO_2_) were detected.

After five weeks in nitric acid, a cassiterite (SnO_2_) patina formed on the 3% Sn alloy, while the 7% Sn sample showed additional cuprite (Cu_2_O).

## 3. Experimental Methods

### 3.1. Material and Ageing Test Configuration

Cultural artifacts were represented using bronze alloys comprising copper with either 3% Sn or 7% Sn and 0.1%–0.5% by mass of other trace materials [36]. Sample discs (12 mm in diameter and 2 mm in thickness) were polished with abrasive papers of 400 to 1200 grade, and then cleaned with 10 vol% sulphuric acid, rinsed with distilled water, and degreased with acetone.

The tested aqueous layers included two solutions of individual acids (H_2_SO_4_ and HNO_3_, each 0.1 M) and a mixed solution containing both acids designed to represent the composition and acidity of acid rain (Table 5). Sample discs were immersed in the solutions for one week, then dried in air for several hours and measured, before being re-submerged for another week and then re-measured; this cycle was repeated a total of five times.

### 3.2. Methods

Electrochemical methods, open circuit potential (OCP) measurements, and electrochemical impedance spectroscopy (EIS) were used to quantify ion transfer inside the surface layers and in the electrolyte. The results allowed the determination of the mechanisms of patina growth, the stages of corrosion and their rates, and also the properties and stability of the patina.

The OCP and EIS measurements were repeated weekly using an Autolab PGStat 20 instrument (Metrohm autolab, Utrecth, The Netherlands). The EIS was conducted using a three-electrode cell comprising an Ag/Ag/Cl reference electrode, a Pt counter electrode, and a bronze working electrode in a solution of 0.1 M sodium sulphate. The OCP values were taken before the EIS measurements. In each case, the stable value reached after some minutes of immersion in the electrolyte was noted. The weekly OCP measurements represent an index of the surface changes as well as the trend in the values.

The impedance frequency was scanned from 75 kHz to 1 Hz at 10 mV. This frequency range allowed the determination of the different electrochemical processes occurring inside the cell (on the surface of the samples and in the double layer), as well as the properties of the patina and the mechanism of its formation. The formation and the characteristics of the patina can be described by equivalent circuits and their elements extrapolated from the data elaboration of EIS. The equivalent circuits were obtained using an Autolab FRA Analyzer (Eco Chemie, Utrecth, The Netherlands) and with an EIS Spectrum Analyzer (Alexander S. Bondarenko and Genady A. Ragoisha, Physico-Chemical Research Institute Belarusian State University). The film resistance (*R*_f_) describes the patina passivity as well as the film capacitance (*C*_f_). The resistance (*R*_ct_) and capacitance (*C*_ct_) of charge transfer relate to the reaction kinetics [31,37]. The resistance of the electrolyte is represented by *R*_e_. The Warburg (*W*) represents diffusion phenomena as well as the patina porosity [5,31,37]; the constant phase element (CPE) related to the capacitance (*Q*) shows the inhomogeneous character of the system, frequently considered as electrode roughness. The *n* index is present only with *Q* and the values are between 1 and 0. This index decrease in its values with the increase of the surface roughness. It can also represent the inhomogeneity porosity related to the Warburg element (*n* close to 0.5), indicating a possible presence of a diffusion mechanism inside the layer [37]. The single circuit element cannot be distinguished, allowing the sums of the resistances (*R*_t_ = *R*_e_ + *R*_f_ + *R*_ct_) and of the capacitances (*C*_t_ = *C*_f_ + *C*_ct_ + *Q*) to be found.

The relative thickness *T* of the patina (single or multilayer) can be evaluated using the following equation [37]:

$$T=1/C_{f}$$ (1)

when the film capacity *C*_f_ is distinguishable from the other capacitance components (*C*_t_) [37].

After five weeks, the patina was characterized by spectrocolorimetry, scanning electron microscopy (HITACHI S 2500 SEM, Hitachi High-Tech, Tokyo, Japan) with energy-dispersive X-ray spectroscopy (EDS, Hitachi High-Tech, Tokyo, Japan), and X-ray diffractometry (XRD Thermo Scientific* ARL X′TRA, Thermo scientific, Waltham, MA, USA). The color morphologies and corrosion layers were associated with the crystalline structure of the patina.

Colorimetric measurements were carried out using a portable spectrophotometer (X-Rite SP64, X-rite, Althardstrasse, Switzerland). The percentage of reflected light (*R*) was measured as a function of the wavelength (nm). The color difference (Δ*E*) between the clean and the corroded alloys was calculated based on the difference between the colorimetric parameters L* (lightness), a* (red–green component), and b^*^ (yellow–blue component) using the following equation [38]:

$$\Delta E=\sqrt{{\left(\Delta L^{*}\right)}^{2}+{\left(\Delta a^{*}\right)}^{2}+{\left(\Delta b^{*}\right)}^{2}}$$ (2)

where Δ*E* is an index of color damage (*i.e*., the change from the color of the natural alloy) used to describe the corrosion. Here, Δ*E*, calculated from the parameters of the clean alloy (time = 0) and after the five weeks of tests, indicates the patina formation. Its progression represents the formation process: fast and deep corrosion processes produce greater color changes as well as a more developed patina.

A Hitachi S 2500 machine (HITACHI S 2500 SEM, Hitachi High-Tech, Tokyo, Japan) equipped with a LaB6 electron source and a scintillation electron photo detector was used to record SEM images of the morphology of the corrosion products. The typical working pressure was 10^−7^ mbar. An XRD procedure was used to characterize the corrosion products, using a Thermo Scientific^*^ ARL X’TRA powder diffractometer (XRD Thermo Scientific* ARL X′TRA, Thermo scientific, Waltham, MA, USA) with the following setup: 40 kV, 40 mA, 2θ scanning from 10° to 80° grade, 0.02 step width, and counting time of 5 s per step.

## 4. Conclusions

Open circuit potential measurements of bronzes with 3% Sn and 7% Sn were recorded at weekly intervals during extended immersion tests in various acid solutions (artificial acid rain (pH = 3.4), 0.1 M sulphuric acid, and 0.1 M nitric acid). In a solution of synthetic acid rain (mixed nitric and sulphuric acids) a more stable patina formed on the 7% Sn bronze than on the 3% Sn sample. Exposure to sulphuric acid alone caused a more protective layer to form on the 7% Sn alloy than on the 3% Sn sample. Both samples showed similar evolutions of open circuit potential when in nitric acid alone; a slightly more stable patina formed on the 3% Sn alloy.

Electrochemical impedance spectroscopy corroborated the OCP measurements by detecting the emergence of a protective patina on the 7% Sn sample immersed in the acid rain solution, while the 3% Sn alloy showed a thick layer that frequently partially dissolved.

Immersion in sulphuric acid led to similar results: the 3% Sn alloy was more damaged than the 7% Sn sample, which showed different, better corrosion resistance. In nitric acid, the 7% Sn bronze formed a less stable patina that frequently partially dissolved. Although a more stable patina formed on the 3% Sn sample, this sample generally showed greater damage than the 7% Sn alloy. This result is in accordance with the OCP measurements. Analysis of the equivalent circuit elements elucidated the various specific corrosion mechanisms occurring on the two alloys.

Colorimetric measurements showed that both alloys changed their natural color owing to the acid rain solution, the 7% Sn bronze more than the 3% Sn sample. The two alloys each produced a layer that altered their original color.

The patina of the 3% Sn sample was also more greatly modified (*i.e*., it showed a greater Δ*E* value) by nitric acid than was that of the 7% Sn sample, corroborating the OCP and EIS results.

Considering the extents of the color changes (Δ*E*), the 3% Sn alloy appeared to be most greatly affected by nitric acid, followed by sulphuric acid and the acid rain solution. The 7% Sn alloy was most aggressively affected by sulphuric acid, followed by the acid rain solution and nitric acid. Finally, the mixture of acids did not correspond to harsher corrosion than that due to the single acids, and the tin content of the bronzes affected how they were attacked by the different acid solutions.

## Figures and Tables

**Table 1.** Equivalent circuits for 3% Sn bronze and 7% Sn bronze immersed in the artificial acid rain solution. The film resistance (*R*_f_); the film capacitance (*C*_f_); the resistance (*R*_ct_) and capacitance (*C*_ct_) of charge transfer; the resistance of the electrolyte (*R*_e_); the Warburg (*W*); the constant phase element (*Q*); and its index *n* (present only with *Q*).

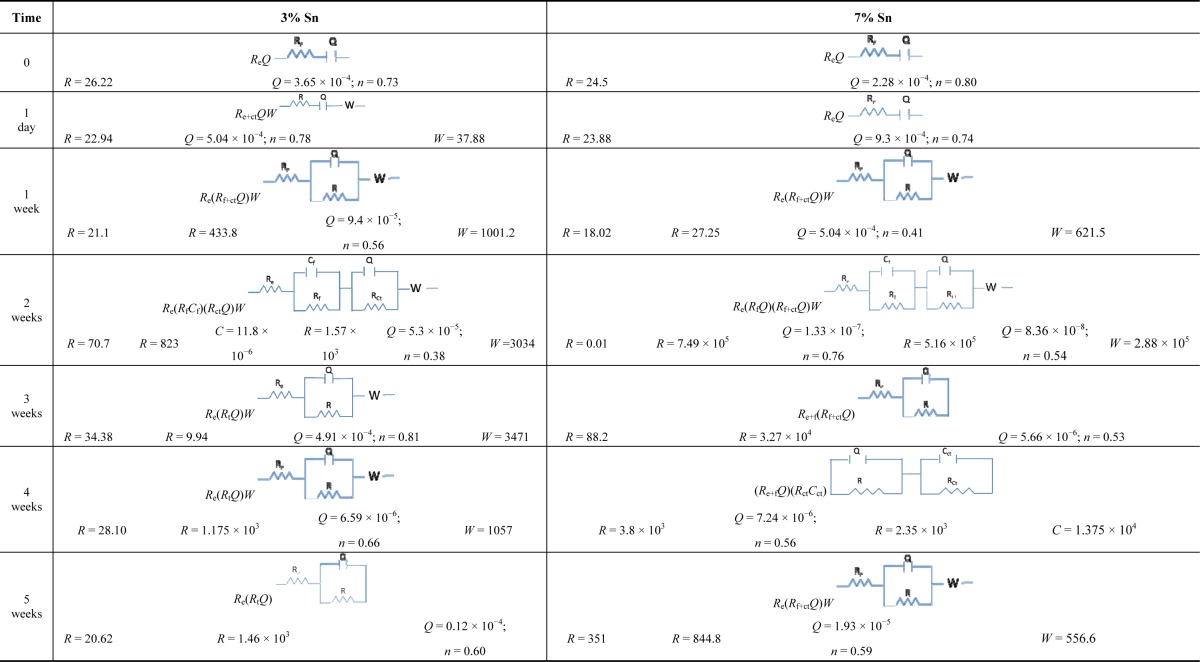

**Table 2.** Equivalent circuits for 3% Sn bronze and 7% Sn bronze immersed in 0.1 M sulphuric acid solution. The film resistance (*R*_f_); the film capacitance (*C*_f_); the resistance (*R*_ct_) and capacitance (*C*_ct_) of charge transfer; the resistance of the electrolyte (*R*_e_); the Warburg (*W*); the constant phase element (*Q*) and its index *n* (present only with *Q*).

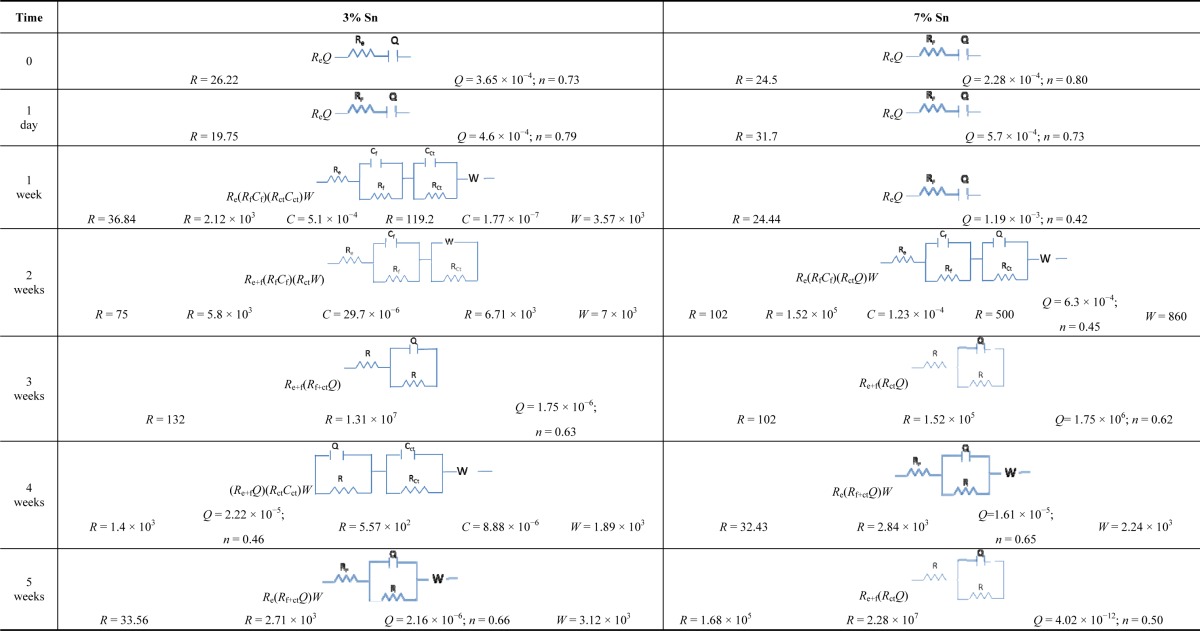

**Table 3.** Equivalent circuits for 3% Sn bronze and 7% Sn bronze immersed in 0.1 M nitric acid solution. The film resistance (*R*_f_); the film capacitance (*C*_f_); the resistance (*R*_ct_) and capacitance (*C*_ct_) of charge transfer; the resistance of the electrolyte (*R*_e_); the Warburg (*W*); the constant phase element (*Q*) and its index *n* (present only with *Q*).

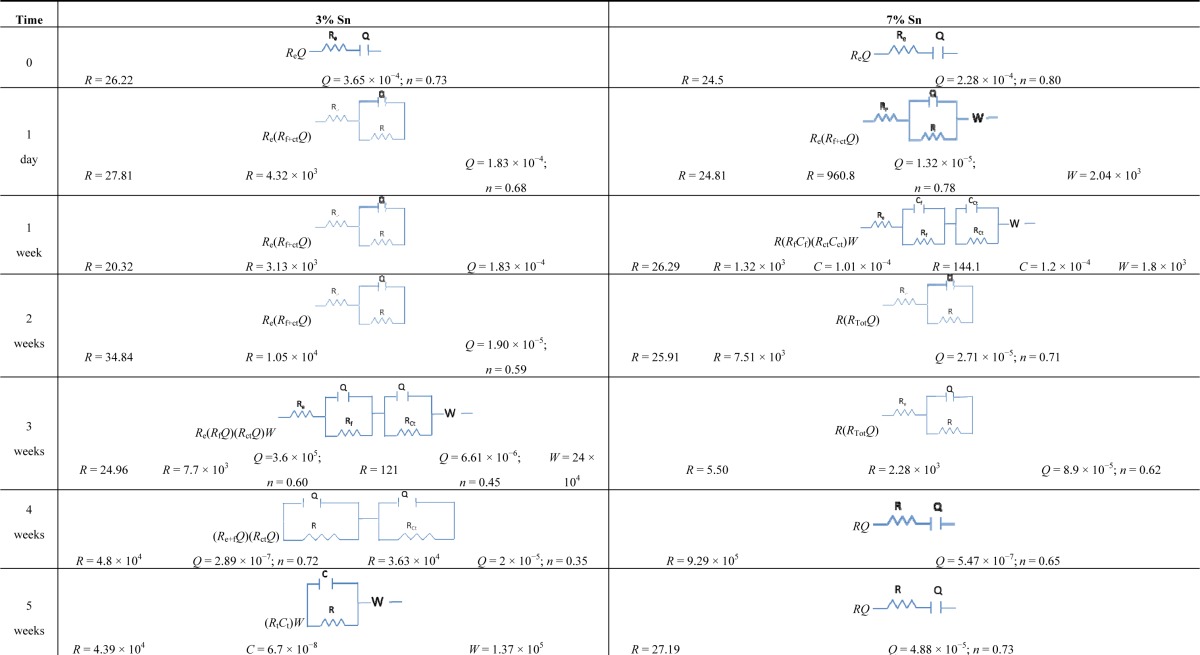

**Table 4.** Color parameters L* (lightness), a* (red–green component), and b* (yellow–blue component) of the bronzes before and after the immersion testing listed along with Δ*E.*

Tests	L*	a*	b*	Δ*E*
3% Sn Time = 0; Clean	83.63	10.35	14.17	–
3% Sn Acid rain	52.08	−1.55	12.08	33.78
3% Sn 0.1 M H_2_SO_4_	49	6.06	−3.35	39.04
3% Sn 0.1 M HNO_3_	42.34	8.13	14.07	41.33
7% Sn Time = 0; Clean	84.07	8.26	14.63	–
7% Sn Acid rain	49.89	1.13	9.43	35.28
7% Sn 0.1 M H_2_SO_4_	50.26	4.13	−3.94	38.79
7% Sn 0.1 M HNO_3_	56.59	9.3	19.48	28.47

**Table 5.** Synthetic acid rain (pH = 3.4) composition.

Component	Density (mg/dm^3^)
H_2_SO_4_ (96%)	31.85
(NH_4_)_2_SO_4_	46.20
Na_2_SO_4_	31.95
HNO_3_ (70%)	15.75
NaNO_3_	21.25
NaCl	84.85
